# Patient enablement requires physician empathy: a cross-sectional study of general practice consultations in areas of high and low socioeconomic deprivation in Scotland

**DOI:** 10.1186/1471-2296-13-6

**Published:** 2012-02-08

**Authors:** Stewart W Mercer, Bhautesh D Jani, Margaret Maxwell, Samuel YS Wong, Graham CM Watt

**Affiliations:** 1 General Practice and Primary Care, Institute of Health and Wellbeing, University of Glasgow, 1 Horselethill Road, Glasgow, UK; 2 School of Nursing, Midwifery & Health, University of Stirling, Stirling, UK; 3 School of Public Health and Primary Care, The Chinese University of Hong Kong, Hong Kong, China

**Keywords:** Patient Enablement, Empathy, General Practice Consultation, Socio-economic Deprivation

## Abstract

**Background:**

Patient 'enablement' is a term closely aligned with 'empowerment' and its measurement in a general practice consultation has been operationalised in the widely used patient enablement instrument (PEI), a patient-rated measure of consultation outcome. However, there is limited knowledge regarding the factors that influence enablement, particularly the effect of socio-economic deprivation. The aim of the study is to assess the factors influencing patient enablement in GP consultations in areas of high and low deprivation.

**Methods:**

A questionnaire study was carried out on 3,044 patients attending 26 GPs (16 in areas of high socio-economic deprivation and 10 in low deprivation areas, in the west of Scotland). Patient expectation (confidence that the doctor would be able to help) was recorded prior to the consultation. PEI, GP empathy (measured by the CARE Measure), and a range of other measures and variables were recorded after the consultation. Data analysis employed multi-level modelling and multivariate analyses with the PEI as the dependant variable.

**Results:**

Although numerous variables showed a univariate association with patient enablement, only four factors were independently predictive after multilevel multivariate analysis; patients with multimorbidity of 3 or more long-term conditions (reflecting poor chronic general health), and those consulting about a long-standing problem had reduced enablement scores in both affluent and deprived areas. In deprived areas, emotional distress (GHQ-caseness) had an additional negative effect on enablement. Perceived GP empathy had a positive effect on enablement in both affluent and deprived areas. Maximal patient enablement was never found with low empathy.

**Conclusions:**

Although other factors influence patient enablement, the patients' perceptions of the doctors' empathy is of key importance in patient enablement in general practice consultations in both high and low deprivation settings.

## Background

The consultation is the core activity of general practice, where important decisions are made by doctors and patients that influence both the use of resources and the likely outcome of patients' problems. Howie and colleagues proposed that the concept of 'enablement' represents the extent to which a patient feels empowered after a medical consultation, in terms of being able to cope with, understand, and manage their illness [1,2]. The Patient Enablement Instrument (PEI) was developed as a means of assessing the outcome of consultations in general practice by measuring the extent to which patients feel better able, as a result of visiting their GP, to cope with and understand their condition and keep themselves healthier [1,2]. The PEI has been widely used in the evaluation of doctors in the UK [3-6] and other countries [7-9]. Patient enablement is distinct from patient satisfaction. Patient satisfaction tends to reflect the extent to which patients' perceptions of delivery of care have been met, while enablement is a complementary but different concept reflecting the achievement of a degree of health literacy and self-care behaviour resulting from improved communication with their GP [3]. Recent preliminary evidence suggests it may also be predictive of longer term health outcomes [10-12].

Many factors may impinge on patient enablement and the outcome of consultations. These potentially include patient factors, consultation factors, and system factors. In terms of *patient factors*, early work on the PEI suggested an influence of age and ethnicity [2,7] and other studies have found effects of patients' stated anxiety levels [8]. *Consultation factors *associated with enablement in primary care suggest the importance of consultation length, and interpersonal continuity of care [2,3], and the doctors' interpersonal aspects include showing an interest in the patients' life, health promotion, and having a positive approach [8]. Associations have also been shown between physician empathy and patient enablement in a range of settings [2,6,11,15-18]. *System factors *(which may arguably include consultation length and continuity of care) also indicate that practice size may be important, with larger practices having lower enablement scores [3].

In areas of high deprivation such as the West of Scotland we have recently reported lower patient enablement in patients with psychological distress in high deprivation areas compared with less deprived areas [16]. A study on factors influencing a shortened version of the PEI also found effects of socio-economic status on enablement, as well as associations with age, ethnicity, continuity, and positive perception of GP communication [15].

Thus a number of factors seem to relate to patient enablement; it is important to elucidate these factors in terms of independent variables in order to understand how enablement can be enhanced. However, one limitation in much of the literature to date is a lack of robust statistical analysis to support conclusions about factors influencing patient enablement. Many of the previous studies have used simple correlations rather than multi-regression analysis, and none have controlled for multi-level effects [19].

## Methods

This study comprised a patient-completed, anonymous 6-page questionnaire collecting a range of details including aspects of the organisation of care, perceived needs, perceived process, and outcome of consultations with the participating GPs.

### Sampling frame

A database containing the mean deprivation scores of all GP practices in the west of Scotland was made available by the Information and Statistics Division of NHS Scotland. Deprivation data was extracted on practices in 4 health board regions in the west of Scotland; Greater Glasgow, Argyll and Clyde, Lanarkshire, and Ayr and Arran. Ethical approval for the study was obtained from each of the 4 health boards. The 'low deprivation' group of practices invited to participate in the study were selected from practices in the lower quartile of deprivation scores for the 4 regions combined. In the high deprivation groupings, practices were selected from those in the lower quartile of the combined deprivation scores of practices in the three health board regions out-with Greater Glasgow, and those in the lower quartile of deprivation scores within Greater Glasgow. This was necessary because of the concentration of severe deprivation within Greater Glasgow.

Because patient enablement scores have been shown to be influenced by practice size [3], the sampling frame was limited to medium-sized Practices (3-4 Partners). Only non-training Practices (i.e., those that are not accredited for training GP Registrars) were included. All medium-sized practices (3 to 4 GP principals) in the upper or lower quartile of deprivation (based on a multiple index-of-deprivation score used nationally) in 4 health board regions in the west of Scotland were mailed letters that explained the details of the study and asked the practice to nominate 1 GP to participate. 26 GPs from 26 Practices agreed to participate in the study, from 70 eligible practices approached across the 4 health board areas, giving an overall recruitment rate of 37% (36% in the high deprivation groups and 38% in the low deprivation groups). We have reported some further details on the sampling frame in previous papers [16,20].

### Patients

Consecutive, unselected patients were asked by the reception staff if they would be willing to complete a questionnaire when they arrived for their consultations. This self-completed, anonymous questionnaire collected details of perceived needs, perceived process, and outcome of consultations with the participating GPs. Individual demographic, socio-economic, and health details were also collected [16,20].

The process of the consultation was assessed using the Consultation and Relational Empathy Measure (CARE Measure), a validated tool which seeks to capture the patient's perception of the doctor's empathetic understanding and action in the consultation [20]. Continuity of care was assessed by asking if the patients were seeing their usual doctor, and if so, how well they felt they knew the doctor, on a 5 point scale as used previously [21]. Consultation length was recorded by the doctor. The outcome of the consultation was measured using the patient enablement instrument (PEI) [1,2].

The questionnaire also recorded age, gender, marital status, children, employment status, and educational level, type of living accommodation, ethnicity, and postcode. The latter was used to calculate individual deprivation scores. General health information included the General Health Questionnaire (GHQ-12) [21], general health over the previous twelve months, and any long-term illness, health problem, or disability. The number and type of chronic diseases was also recorded as described previously [22].

The reason for making the consultation ('new problem', 'long-standing problem' or 'both new and old problems'), how many problems the patient wished to discuss, confidence that the doctor would be able to help (patient expectations), whether the patient hoped to receive a new prescription, and the GHQ-12 and socio-economic and demographic details were all recorded before the consultation [16]. A complex consultation was defined as one in which a patient wanted to discuss a psychological or social problem (with or without a physical problem) as previously explained [16]. GHQ-caseness (meaning significant psychological distress) was defined as having a cut-off of 4 or above as previously described [16,20]. The other items relating to the process and outcome of care, and general health were completed straight after the consultation. The completed questionnaires were then collected in a sealed box at the reception area.

The patient response rate to the questionnaire overall was 70% (70% in high deprivation group, 71% in low deprivation group). Although data was not collected on the 30% of consulting patients who chose not to participate in the study, we have reported the distribution of participating patients per practice, as a percentage of the distribution of deprivation (in quartiles) of all patients registered with that practice and shown that here was a reasonably equitable spread of deprivation scores of participating patients, suggesting that the patients who declined to participate were not substantially skewed towards the most deprived end of the spectrum [20].

### Data analysis

Important patient, consultation, and system variables collected in the patient survey were treated as potential confounding factors for enablement and were evaluated for their association with PEI by use of multi-level modeling [19] that took into account the hierarchical nature of the data (patients within GPs). Because of the skewed nature of the PEI, we carried out the analysis by way of binary logistic regression, with PEI scores categorized as below average versus average or above. Univariate analysis was done first for all potential confounding factors. Those factors with P < 0.25 in univariate analysis were then analyzed by multivariate analysis with use of a forward stepwise selection strategy. In general, the process added the most significant confounding factor to the model at each step and continued until all confounding factors that made a significant (P < 0.05) contribution were in the model.

The models are fitted by the method of Markov Chain Monte Carlo (MCMC) algorithm of MLn for Windows software package (Version 2.02, Institute of Education, University of London, U.K.). The Deviance Information Criterion (DIC) diagnostic statistic, which is a generalization of the Akaike Information Criterion (AIC), is used to assess the statistical significance of the estimates at 5% level of significance.

## Results

**Figure 1:**
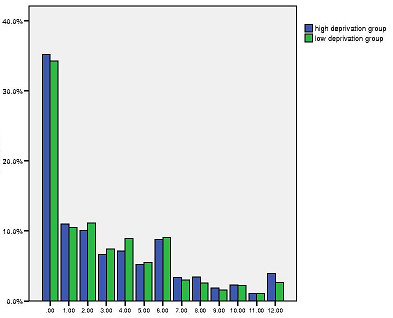
**Distribution of PEI scores in high and low deprivation groups**. X-axis: Enablement Score- PEI. Y-axis: Percentage.

The analysis involved data collected from 3,044 patients (1,966 patients in the high deprivation group, and 1,078 patients in the low deprivation group). The mean patient age was 43.4 years and 46.5 years, in the high and low deprivation groups respectively. Female patients made up 61% of the high deprivation group and 65% of the low deprivation group. Full details of the two groups in terms of patient characteristics (see table 1) and consultation quality markers (including enablement scores) have been published previously [16,20]. The overall mean enablement score was 3.0 (SD 3.36, n = 2471) with a median of 2.0. Mean PEI scores were similar in high and low deprivation areas; high deprivation 3.1, (SD 3.44, n = 1531), low deprivation group 3.0, (SD 3.21, n = 940). The median for both areas was 2.0. The distribution of PEI scores in high and low deprivation settings are shown in Figure 1. As can be seen, the scores were not normally distributed.

**Table 1:** Characteristics of Patients living in high and low deprivation areas

Characteristics	Categories	Most Deprived Areas n (%)	Least Deprived Areas n (%)	*P *value
Age	< 30 years	411 (23.5%)	176 (17.8%)	< 0.001

	30-60 years	972 (55.7%)	560 (56.5%)	

	> 60 years	363 (20.8%)	256 (25.7%)	

Sex	Female	1176(65.3%)	615 (61%)	0.024

Emotional distress (GHQ caseness)	4 or more	652 (41.3%)	273 (28.6%)	< 0.001

Number of long-term conditions	0	485 (24.7%)	271 (25.2%)	0.008

	1	491 (25%)	320 (29.7%)	

	2	387 (19.7%)	223 (20.7%)	

	3 or more	599 (30.5%)	262 (24.3%)	

General Health	Very good	206 (11.3%)	166 (16.1%)	< 0.001

	Good	471 (25.9%)	375 (36.5%)	

	Fair	665 (36.6%)	339 (33%)	

	Bad	383 (21.1%)	122 (11.9%)	

	Very Bad	94 (5.2%)	26 (2.5%)	

In view of this non-linear distribution of PEI scores, these scores was analysed as a binary outcome (PEI score below 3.0 or 3.0 and above) thus overcoming assumptions of linearity in the analysis. We carried out binary logistic regression with PEI score as the dependant variable and a score of less than 3 as the reference category. Univariate analysis showed 7 factors to be significantly associated with PEI scores; (Table 2).

**Table 2:** Factors associated with patient enablement: univariate analysis n = 3044

Variable	Categories	Odds Ratio	*P *value
Confidence that doctor will be able to help	Not confident	1	
	
	Moderately confident	3.11 (1.46-6.62)	0.0033
	
	Very confident	1.89 (0.89-4.02)	0.098
	
	Completely confident	4.29 (2.06-8.97)	0.0001

Reason for seeing doctor	New problem	1	
	
	Longstanding problem	1.18 (0.96-1.44)	0.10
	
	Both new and old problems	0.76 (0.62-92)	0.006
	

Complexity of problem	Non complex (physical)	1	
	
	Complex (psychosocial)	0.78 (0.65-0.93)	0.0064

Patients' perception of GP empathy (CARE Measure)	Less than 40	1	
	
	40 or more	2.13 (1.87-2.43)	0.00001

Emotional distress (GHQ-caseness)	Less than 4	1	
	
	4 or more	0.50 (0.41-0.62)	0.00001
	

General health over last 12 months	Very good	1	
	
	Good	0.91 (0.60-1.38)	0.65
	
	Fair	0.54 (0.37-0.77)	0.0083
	
	Bad	0.33 (0.23-0.48)	0.00001
	
	Very Bad	0.14 (0.08-0.27)	0.00001

Number of long term conditions	0	1	
	
	1	1.06 (0.8-1.39)	0.688
	
	2	0.86 (0.65-1.13)	0.269
	3 or more	0.63 (0.49-0.81)	0.0002

1. Expectation before the consultation

2. Reason for seeing the doctor (new or long-standing problem)

3. Complexity of problem (physical or psychosocial +/- physical)

4. Perception of the GPs empathy (CARE score)

5. Psychological distress (GHQ-caseness)

6. General health over the last 12 months

7. Multimorbidity (number of long-term conditions)

However, after multilevel multivariate analysis, only 4 factors remained independently associated with PEI score; (see Table 3)

**Table 3:** Factors associated with patient enablement: multi-variate analysis n = 3044

Variable	Categories	Odds Ratio	*P *value
Reason for seeing doctor	New problem	1	
	
	Long standing problem/Both new and long standing problem	0.61 (0.08-0.76)	0.00002

Perceptions of GP empathy (CARE Measure)	Less than 40	1	
	
	40 or more	2.29 (0.29-2.73)	0.00001

Emotional distress (GHQ-caseness)	Less than 4	1	
	
	4 or more	0.56 (0.07-0.78)	0.0004

Multi-morbidity (number of long term conditions)	0	1	
	
	1	1.16 (0.14-1.58)	0.350
	
	2	0.89 (0.1-1.33)	0.577
	
	3 or more	0.67 (0.08-0.94)	0.02

1. Reason for consulting (new or long-standing problem/both)

2. Psychological distress (GHQ-caseness)

3. Multimorbidity

4. Perception of the GPs empathy (CARE Measure score)

To determine whether these independent predictors of enablement operated in patients from both high and low deprivation areas, interaction effects of deprivation group on each variable were examined. The effect of the 4 independent predictors of enablement and the influence of deprivation are detailed below.

### Reason for consulting

Patients who were consulting about a new problem had higher PEI scores (i.e., a higher percentage scoring 3 and above) than those consulting for a long-standing problem (or both new and long-standing). There was no significant interaction effect with deprivation (results not shown).

### Psychological distress

**Figure 2:**
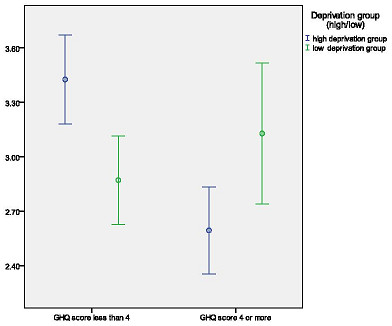
**Effect of emotional distress on patient enablement in high and low deprivation groups**. X-axis: Emotional distress. Y-axis: 95% Confidence interval for mean PEI-enablement score.

Patients who had significant psychological distress (GHQ caseness) had lower PEI scores (i.e., a higher percentage scoring 3 and above) than those who did not have caseness. However, there was a highly significant interaction effect with deprivation, which indicated that the effect of psychological distress on PEI score was only apparent in the high deprivation group. Please see Figure 2 which shows the interaction between GHQ caseness and deprivation status.

### Number of long-term conditions/general health

Patients who had 3 or more long-term conditions had lower PEI scores (i.e., a lower percentage scoring 3 and above) than those with less than 3 conditions. There was no significant interaction effect with deprivation (results not shown).

When the number of long-term conditions (multimorbidity) variable in the multi-variate model was replaced by self-reported general health over the last 12 months, a similar finding was observed - patients with worse general health had lower PEI scores. Again, there was no significant interaction effect with deprivation (results not shown).

The number of long-term conditions and general health were not included in the model together because of a significant association between these two variables. The association between number of long-term conditions and general health was highly significant (Pearson's r = 0.423, p < 0.0001).

### Patients' perceptions of GP empathy (CARE score)

Patients who perceived their GP to be empathic (above average CARE measure score in the current study) had higher PEI scores (i.e., a higher percentage scoring 3 and above) than those who scored their GP as having average or below average empathy. There was no significant interaction effect with deprivation (results not shown).

Further examination of the relationship between GP empathy and patient enablement showed that full enablement (maximal PEI score of 12; n = 82) never occurred when GP empathy was low (lower quartile of CARE Measure scores), whereas 63.4% (n = 52) of full enablement consultations occurred when GP empathy was high (upper quartile of CARE Measure scores) and 36.6% (n = 30) when GP empathy was average (second and third quartiles of CARE measure scores). The findings were similar in both high and low deprivation areas (results not shown). Thus, although high empathy was not always associated with high enablement (presumably due to the other factors that negatively influence enablement such as emotional distress, multimorbidity, etc), low empathy was always associated with low enablement, suggesting that GP empathy is a basic pre-requisite for patient enablement.

## Discussion

The present analysis explored the factors associated with patient enablement in general practice consultations in deprived and affluent areas, using robust statistical methods to control for multi-level interactions (cluster effects). Four factors independently influenced enablement; three were common to both deprived and affluent areas - type of problem (new or long-standing), multimorbidity (or poor general health) and perception of the GPs' empathy - and one factor was specific for patients living in deprived areas (a negative effect of psychological distress on enablement).

### Relationship to literature

Patient expectation before the consultation was assessed by a single item question, 'how confident are you that the doctor will be able to help you today?' Although this is not a validated measure, it has been used previously in research on patient expectation, and we have found it to be related to patient enablement in patients attending complementary therapists [12]. However, as far as we are aware, this is the first study that has examined the effect of patients' expectations on enablement in general practice consultations. Although expectation did have an association with enablement in the univariate analysis, this association did not remain after multi-variate analysis.

Reason for encounter (seeing the doctor for a new or long-standing problem) did emerge as a significant independent factor in enablement. This is an important new finding as patients in deprived areas consult more frequently with chronic conditions [16]. Although in the present study we did not find a difference in overall PEI scores between high and low deprivation areas, in agreement with the original work on enablement [2], a more recent large study (using a shortened version of the PEI) has reported lower enablement in patients of lower socio-economic status, and one explanation of this could be reason for consultation [15].

Enablement was also independently negatively influenced by psychological distress, but this effect was only seen in the high deprivation areas. We have previously reported a similar association between complexity of consultation (patients wishing to discuss psychological or social problems plus or minus physical problems) and enablement in deprived areas in our original analysis of this study [16], but this previous work was descriptive and not subject to multilevel multivariate analysis. The robust association with GHQ-caseness shown in the present analysis is important; given the higher prevalence and severity of mental health problems in patients living in deprived areas, and the fact that much mental illness goes undetected in general practice consultations [23].

Enablement was also associated with patients' self-reported general health over the last 12 months; patients with poorer long-term health reported less enablement at consultation. This again has implication for enablement in deprived areas, as self-reported general health is much lower in deprived areas compared with affluent areas and appears to be an important predictor of mortality. The presence of a long-term condition had an additional negative influence of enablement, and again patients in deprived areas have more long-term conditions and multiple morbidity [16].

Given these negative 'pressures' (poorer general health, poorer mental health, more multimorbidity, more consultations for chronic problems) on enablement in patients living in deprived areas, the fact that enablement scores overall were not different in the high and low deprivation settings requires further exploration, but implies that the patients in such areas with better health and consulting with acute problems of a non-psychological nature are being enabled more than their counterparts in the more affluent areas. One explanation of this may relate to receiving a desired prescription, as previous work has shown that patients who expected and received a prescription at consultation reported higher enablement scores [2].

Patient perception of the GPs empathy (assessed with the CARE Measure) was positively related to enablement. GP empathy emerged as an essential pre-requisite for enablement in both high and low deprivation settings; although high empathy did not guarantee high enablement, enablement never occurred with low empathy. Consultation length and continuity of care (knowing the doctor well) were not related to enablement in the current study. Previous work did find a weak positive association between enablement and consultation length and continuity of care [2]. Two factors may explain this; firstly the current study was substantially smaller than the previous study (and may thus have lacked the power to show weak associations), and secondly the statistical analysis in the current study was more sophisticated than in the earlier work.

We have found similar results using the same measures in several studies in different settings [12,13,17,18]. The CARE Measure is being widely used in the NHS in the UK [20,24,25], and internationally and these findings add validity to the measure - which is a measure of the process of the consultation - in terms of its importance in patient-reported outcomes (in this case patient enablement).

### Strengths and weaknesses

As reported previously, a major strength of the present study is that it achieved a good response rate and that that even the most socio-economically deprived patients within the poorest areas were represented in the study sample [16,20]. The study was also one of the most comprehensive so far conducted in terms of the range of possible factors at patient, consultation, and system level that could have an influence of patient enablement.

The study also had limitations. Although large in patient numbers, it was relatively small in terms of the number of GPs who participated (n = 26). Furthermore, we cannot be sure that the GPs who volunteered to part in the study are representative of GPs working in such localities. The study did not attempt to link GP empathy or patient enablement to health outcomes and further work is required on this, though we have published pilot work which suggests a link between empathy, enablement and outcomes [12].

## Conclusions

The patients' perceptions of the doctors' empathy is of key importance in patient enablement in general practice consultations in both high and low deprivation settings. Enablement is lower in patients with multimorbidity (of 3 or more conditions) and for those consulting about a long-standing problem. In deprived areas, psychological distress has an additional negative influence. Ways of supporting and improving practitioner empathy may be crucial in enhancing patient enablement, especially in high deprivation areas, where the burden of multimorbidity, mental illness, and poor health is greatest.

## Competing interests

The authors declare that they have no competing interests.

## Authors' contributions

SWM, MM and GCW were involved with the project conception, design and implementation. SW (Samuel Wong) and BJ helped with analysis and interpretation of findings along with the other authors. All authors contributed towards preparing and revising the manuscript for the article. All authors read and approved the final manuscript.

## Pre-publication history

The pre-publication history for this paper can be accessed here:

http://www.biomedcentral.com/1471-2296/13/6/prepub
